# Glances at the Hospitals

**Published:** 1902-02-15

**Authors:** 


### GLANCES AT THE HOSPITALS.

### BRISTOL ROYAL INFIRMARY.

The ordinary income was £10,840, and the ordinary expenditure was £14,925. At the end of the previous year there was due to the treasurer a balance of £13,000, and as much of this adverse balance was caused by expenditure on building operations; it was very properly decided to sell out stock to the extent of £8,532. The governors further point out that during the last ten years they have spent £19,000 on improvements and extensions, exclusive of ordinary repairs, and that although they have thereby decreased their permanent income, they have increased the efficiency of the hospital and the comfort of the patients. We believe this to be an enlightened view of the situation, as an infirmary should mainly rely on its annual subscriptions, and when the invested funds become too large, these annual subscriptions are more likely to diminish than otherwise. It is encouraging to note that the workpeople's contributions amounted to £1,764. The infirmary contained, on January 1st, 204 patients, and during the year 2,782 were admitted, making a total of 2,986. Of these 2,393 were cured or relieved; 188 died, 9 were brought in dead, and 192 remained under treatment. The average daily number resident was 201, and the average duration of each patient's stay in the infirmary was 26 days. The cost of each bed occupied was £59 14s., and the cost of each in-patient was £4. An analysis of the more important medical and surgical cases is given. Of 22 cases of phthisis, 18 were cured or relieved and 4 died. We believe the time is fast approaching when consumptive patients will not be received into general infirmaries except when such infirmaries are also medical schools, and then special provision must be made for the treatment of phthisis. Of 99 cases of pneumonia 88 were cured or relieved, and 11 died. Of 76 cases of typhoid 65 were cured and 11 died. A total of 1,506 operations were performed during the year, and of these 1,392 were cured or relieved, 52 died, and 62 remained under treatment. A good anæsthetic table is given, from which we learn that chloroform was administered 728 times, chloroform and ether mixed 69 times, ether 181 times, and nitrous oxide and ether 208 times, and nitrous oxide alone 266 times. Cocaine and anestile were administered for slight operations.

### BIRMINGHAM GENERAL HOSPITAL.

THIS report is entirely medical, there being no statement by the governors concerning the financial position of the institution. At the beginning of the year there were in residence 262 patients; 4,473 were admitted during the year, making a total of 4,735 under treatment. Of these 2,994 were discharged cured, 608 relieved, not relieved 220, removed and transferred 187, and died 440. Remaining in the hospital 286. On looking over the medical tables we find that 183 cases of enteric fever were admitted. Of these 163 recovered and 20 died. Of 42 cases of rheumatic fever 25 recovered, 16 were relieved, and 1 was not improved. There were 13 cases of diphtheria with 2 deaths. In the gynæcological wards 195 patients were treated to a termination, and the results show that 152 were cured, 20 relieved, 9 not relieved, 3 left the hospital of their own accord, and 11 died. The operations table is unusually well arranged, and it shows at a glance the results of all the operations performed during the year, and an extra column gives remarks on the results where comment is required. From the anæsthetic table we learn that ether was given 580 times alone, and 322 times in combination with other agents, chloroform 445 times, A.C.E. mixture 190 times, and nitrous oxide 90 times. Cocaine and eucaine were used locally and with great success during the removal of half of a huge goitre. The operation lasted 2 hours. There were 2 deaths during anæsthetisation, chloroform being the agent in both instances. One was a case of empyema, and the patient was subject to attacks of syncope and dyspnœa, which had almost proved fatal on two previous occasions. The other was the case of an apparently healthy man, but the post-mortem examination revealed fatty and dilated heart. He struggled violently when being chloroformed, and, like the other case, died before the anæsthetisation was complete. These cases were both unhealthy subjects; but they would seem to illustrate the old saying, that any patient when being put under chloroform is never quite safe until the sympathetic is paralysed, hence deaths occur before complete insensibility has been produced, and no doubt this was the reason that the old chloroformists pushed on the process of anæsthetisation. After the use of ether, lobar pneumonia occurred in two cases, broncho-pneumonia in one case, and bronchitis and cough in many.

### CARDIFF INFIRMARY.

THE income was £7,804 and the expenditure was £8,437, leaving a balance of £633 against the hospital. The number of available beds had to be reduced from 152 to 120, as the committee were compelled to close one of the wards from lack of funds. This is to be deplored, but it does not seem that the committee had any alternative. The general public and the workpeople themselves must see that the hospital is not crippled by want of money. The daily average number of patients resident in the infirmary was 111, and the average number of days in the infirmary was 26 per patient, an average lower by two days than that of the previous, but still somewhat high. A total number of 1,508 were treated during the year. Of this number 711 were discharged cured, 495 relieved, 110 left the institution for various reasons, 86 died, and 106 were under treatment on the date of the report. The average cost per bed occupied was £63 15s. It is a curious omission, but no medical statistics are given in the report.

